# Comparison of multispectral singlet oxygen luminescence dosimetry and singlet oxygen explicit dosimetry in artificial phantom

**DOI:** 10.1117/1.JBO.30.S3.S34115

**Published:** 2025-12-17

**Authors:** Weibing Yang, Baozhu Lu, Madelyn Johnson, Dennis Sourvanos, Hongjing Sun, Andreea Dimofte, Vikas Vikas, Robert H. Hadfield, Brian C. Wilson, Timothy C. Zhu

**Affiliations:** a University of Pennsylvania, Department of Radiation Oncology, Philadelphia, Pennsylvania, United States; b University of Pennsylvania, School of Dental Medicine, Department of Periodontics, Philadelphia, Pennsylvania, United States; c University of Pennsylvania, Department of Bioengineering, Philadelphia, Pennsylvania, United States; d University of Glasgow, James Watt School of Engineering, Glasgow, United Kingdom; e University Health Network/University of Toronto, Department of Medical Biophysics, Toronto, Ontario, Canada

**Keywords:** photodynamic therapy dosimetry, singlet oxygen, singlet oxygen explicit dosimetry, multispectral singlet oxygen dosimetry, benzoporphyrin derivative

## Abstract

**Significance:**

Direct detection of singlet-state oxygen (O12) is a critical objective in Type II photodynamic therapy (PDT) due to its pivotal role in mediating therapeutic effects. Although multispectral singlet oxygen dosimetry (MSOLD) has demonstrated the capability to detect O12 luminescence both *in vitro* and *in vivo*, there remains no standardized method for accurately quantifying reactive singlet oxygen, [O2]rx1, based on these measured signals. By contrast, the singlet oxygen explicit dosimetry (SOED) model offers a robust framework for calculating [O2]rx1. Demonstrating that O12 luminescence obtained through MSOLD can reliably quantify [O2]rx1, as achieved by the SOED model, is essential for advancing the accuracy and applicability of PDT dosimetry.

**Aim:**

We aim to evaluate the accuracy and reliability of MSOLD in quantifying O12 concentrations from measured O12 luminescence in benzoporphyrin derivative (BPD)-mediated PDT. The performance of MSOLD is assessed by comparing its results with those derived from the SOED model.

**Approach:**

A continuous-wave 690 nm laser was used to excite a nanoparticle formulation of BPD, tradename Visudyne^®^ in methanol at varying concentrations (2 to 6  mg/L). The singlet oxygen luminescence was measured using an InGaAs spectrometer and analyzed using a singular value decomposition algorithm. Near-infrared singlet oxygen emission at ∼1270  nm was extracted as O12 luminescence. Real-time singlet oxygen spectra were collected over 900 s using a 1.5 mm diameter fiber optic. Ground-state oxygen concentration was measured with a commercial oxygen probe, photosensitizer concentration was determined with a custom-made contact probe, and photon fluence rate was assessed with an isotropic detector. [O2]rx1 was then calculated based on the SOED model.

**Results:**

The extracted singlet oxygen (O12) luminescence exhibited clear concentration-dependent trends, with higher BPD concentrations producing stronger O12 luminescence. Over time, the O12 luminescence decayed due to photosensitizer bleaching. In addition, a strong linear correlation was observed between the O12 luminescence measured via MSOLD and the reactive oxygen species (ROS) concentrations calculated using the SOED model. We also investigated the impact of tissue optical properties on singlet oxygen luminescence detection and developed correction factors to account for their variations.

**Conclusions:**

We demonstrate that singlet oxygen (O12) detected through MSOLD can reliably quantify ROS concentrations in BPD-mediated PDT with accuracy comparable to the SOED model, which requires separate measurements of light fluence rate, photosensitizer concentration, and oxygen availability, followed by modeling to estimate the amount of reactive singlet oxygen. By contrast, MSOLD can also be a more cost-effective, simpler, and faster alternative to SOED as it directly measures the singlet oxygen luminescence to quantify reactive singlet oxygen. Under appropriate correction for tissue optical properties, MSOLD presents a promising, robust, and direct dosimetry solution for clinical PDT applications.

## 1 Introduction

Photodynamic therapy (PDT) is a minimally invasive treatment modality that has gained significant attention for its application in oncology, dermatology, and other medical fields. PDT involves the administration of a photosensitizing agent, which preferentially accumulates in target tissues, followed by irradiation with light of a specific wavelength that corresponds to the absorption peak of the photosensitizer. This interaction produces reactive oxygen species (ROS), particularly singlet oxygen (O12), which induce cytotoxic effects leading to cell death, vascular damage, and an immune response against the treated area.^1^^–^^4^

The effectiveness of PDT is contingent on three key components: the photosensitizer and its concentration in target tissue (e.g., tumor), the light fluence, and the tissue oxygenation. Through the interaction of these components, the generation of singlet oxygen (O12) is considered the pivotal cytotoxic agent responsible for the therapeutic effects observed in PDT with Type II photosensitizers. There are several techniques that have been developed to determine the concentration of O12, such as singlet oxygen explicit dosimetry (SOED),^5^^,^^6^ singlet oxygen luminescence dosimetry,^7^^,^^8^ and time- and wavelength-resolved spectroscopy.^9^^,^^10^ Multispectral singlet oxygen dosimetry (MSOLD) is an emerging technique capable of directly detecting the O12 luminescence, which can be associated with the concentration of singlet oxygen (O12). MSOLD detects the weak near-infrared luminescence at ∼1270  nm when singlet oxygen returns to its ground state. However, this signal alone is insufficient to quantify O12 during PDT because it is affected by variations in the experimental setup, detector sensitivity, fiber optics, tissue optical properties, and other system-related factors. To date, there have been several reports using MSOLD detailing how the O12 luminescence has been used as the dosimetry metric to determine PDT dose for an *in vivo* mouse study.^11^^,^^12^ In this study, we demonstrate that the MSOLD signal in benzoporphyrin derivative (BPD)-mediated PDT exhibits a linear correlation with singlet oxygen (O12) concentration, as validated by comparison with the well-established SOED model, which has proven to be a reliable predictor of PDT treatment outcomes *in vivo*.^13^ SOED determines the O12 concentration by explicitly modeling the photophysical and photochemical pathways that generate O12, using fluence rate, photosensitizer concentration, and oxygenation. We also found out that both MSOLD and O12 luminescence can be impacted significantly by the tissue optical properties, as assessed in phantoms. To translate MSOLD toward clinical online dosimetry, the incorporation of correction factors is essential because the detected MSOLD signal is strongly influenced by the optical properties of the surrounding tissue. Variations in absorption and scattering can alter light transport and therefore affect the accuracy of the measured PDT dose. MSOLD alone is not sufficient to fully account for these complexities, particularly in heterogeneous clinical environments. By integrating appropriate correction factors and combining complementary optical techniques, we aim to improve the robustness and accuracy of online dosimetry measurements. The correction factor (CF) accounting for variations in tissue optical properties is then developed to support the long-term goal of enabling reliable MSOLD-based dosimetry in future clinical applications.

The nanoparticle formulation of BPD (Visudyne^®^) is a second-generation, Type II photosensitizer that generates singlet oxygen exclusively, in contrast to Type I photosensitizers. It has been extensively studied for its effectiveness in PDT.^14^ BPD’s strong absorption at ∼690  nm allows it to be activated by light within the optimal therapeutic window, where tissue penetration is maximal. BPD’s pharmacokinetics, including rapid clearance from normal tissues and selective accumulation in malignant cells, make it a promising agent for targeting various cancers. Upon activation by light, BPD generates ROS, including singlet oxygen, which induces cytotoxic effects, leading to the destruction of cancerous cells.^15^ The ability of BPD to preferentially localize in tumor tissue further enhances its therapeutic efficacy and has led to its widespread use in both preclinical and clinical PDT applications.^16^

In this study, we conducted experiments using a 690 nm continuous-wave laser to excite BPD in a liquid phantom and detected the singlet oxygen spectrum with an Avantes spectrometer (AvaSpec–NIR256–1.7–EVO, Avantes, Lafayette, Colorado, United States) (900 to 1700 nm wavelength range). The extracted O12 luminescence from MSOLD was then compared with the calculated concentrations derived from the SOED model. Correction factors (CFs) were developed to account for variations in tissue optical properties. The results demonstrated consistent agreement, validating that the O12 luminescence measured via MSOLD serves as a robust and straightforward dosimetry solution for clinical PDT applications.

## 2 Material and Methods

### 2.1 Multispectral Singlet Oxygen Dosimetry (MSOLD)

MSOLD is an emerging technique that enables the direct detection of singlet oxygen (O12) generated during PDT. It measures the weak luminescence emitted near 1270 nm within the 1200 to 1600 nm detection window, corresponding to the quantum transition from the excited triplet state to the ground state of molecular oxygen. Due to the extremely short lifetime of O12 (∼45  μs^17^), its signal is inherently difficult to detect and has only recently become measurable *in vivo* through MSOLD. Liquid phantoms (10 mL) containing BPD in methanol with 0.6% Nutrilipid^®^ (20%, B. Braun Medical Inc., Bethlehem, Pennsylvania, United States) ($\mu_{s}^{\prime }=8\;{\mathrm{cm}}^{-1}$ at 690 nm) and 0.002% Black India ink (Black India, Higgins Inc., Leeds, Massachusetts, United States) ($\mu_{a}=0.1{\;\mathrm{cm}}^{-1}$ at 690 nm)^18^ were prepared with BPD concentrations 2, 3, 4, 5, and 6  mg/L. A systematic study was also conducted to investigate how variations in $\mu_{a}$ and $\mu_{s}^{\prime }$ of the liquid phantoms affect singlet oxygen luminescence detection. Black India ink and Nutrilipid^®^ were used to simulate the absorption coefficient ($\mu_{a}$) and reduced scattering coefficient ($\mu_{s}^{\prime }$), respectively.

A stock solution of BPD (0.5  mg/mL) was prepared by dissolving Visudyne^®^ powder in water. The BPD stock solution was then diluted with methanol for the experiment. To minimize methanol evaporation during the experiments, the top of each liquid phantom was sealed with transparent plastic wrap. The tip of the fiber detector was positioned in close contact with the plastic wrap to maximize signal strength and ensure consistent results across all measurements.

**Fig. 1:**
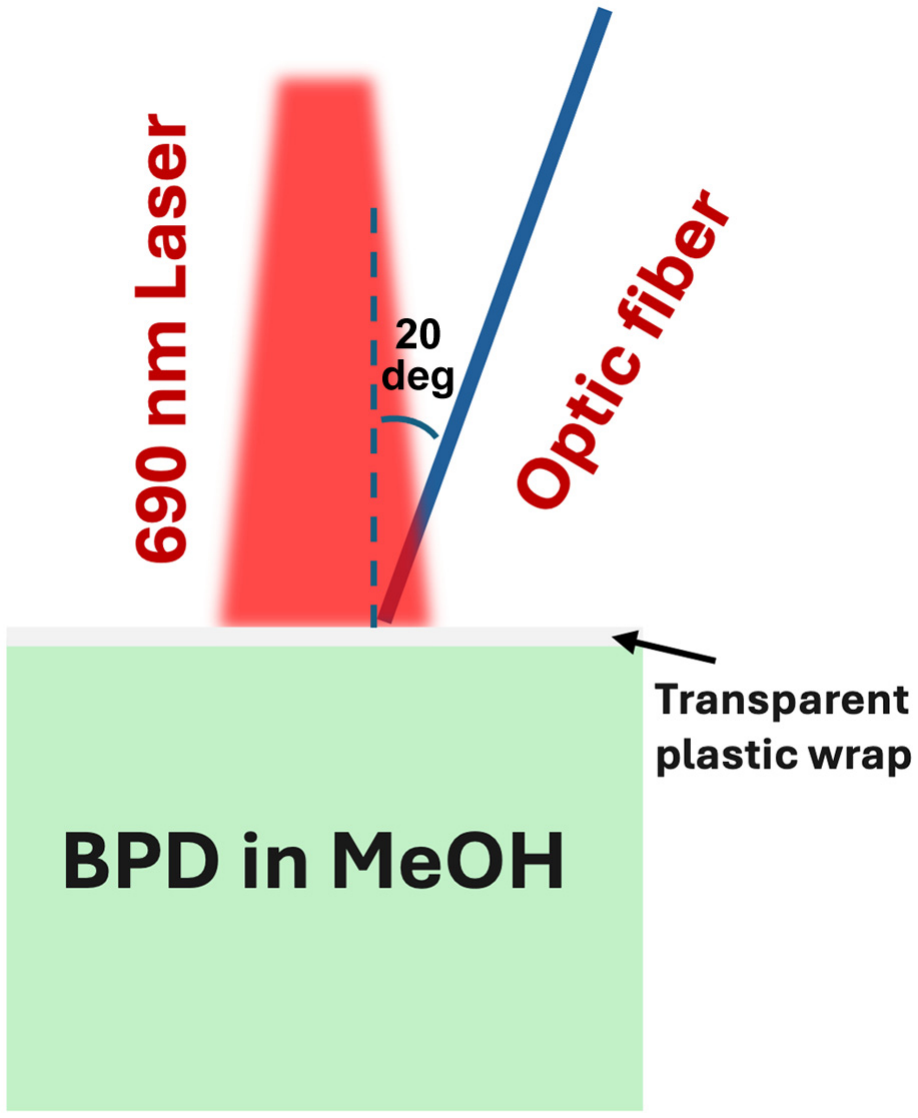
Schematic of experimental setup showing photodynamic therapy on a liquid phantom in methanol, with the laser illuminating top-down and probes positioned on the side of the beam.

The experiment utilized a 690 nm continuous-wave laser (BWF5-690-8-600-0.37, B&W Tek, Newark, Delaware, United States) with a calibrated fluence rate of $850\;\mathrm{mW}/cm^{2}$ with a $1\;{\mathrm{cm}}^{2}$ spot size on the surface of the liquid phantom measured using a power meter (LM-10 HTD, Coherent Inc., California, United States). The phantom is $10\;{\mathrm{cm}}^{2}$ with 1 cm depth and made of black plastic that does not reflect light; thus, boundary effects are negligible. A 1.5 mm core, low-OH flat-cut optical fiber (FT1500EMT, Thorlabs Inc., Newton, New Jersey, United States) with a numerical aperture (NA) of 0.39 was coupled to the InGaAs spectrometer equipped with a 500  μm slit to collect the O12 spectrum in the 1200 to 1600 nm range. Figure 1 illustrates the schematic of the experimental setup, where a transparent plastic wrap is placed between the optical fiber and the liquid phantom; the transparent plastic wrap placed between the contact probe and the liquid phantom does not affect the measurement of optical properties. To maximize the measured singlet oxygen luminescence, the optical fiber tip is positioned at the surface of the phantom. The BPD concentration is measured via a contact probe setup (custom-made), which includes the spectroscopy system equipped with a charge-coupled device (CCD) array for detecting BPD phosphorescence at 690 nm.^19^ The collected 690 nm luminescence was analyzed using MATLAB to isolate the BPD phosphorescence and calculate the BPD concentration by comparing it to the luminescence measured prior to PDT.^20^

**Fig. 2:**
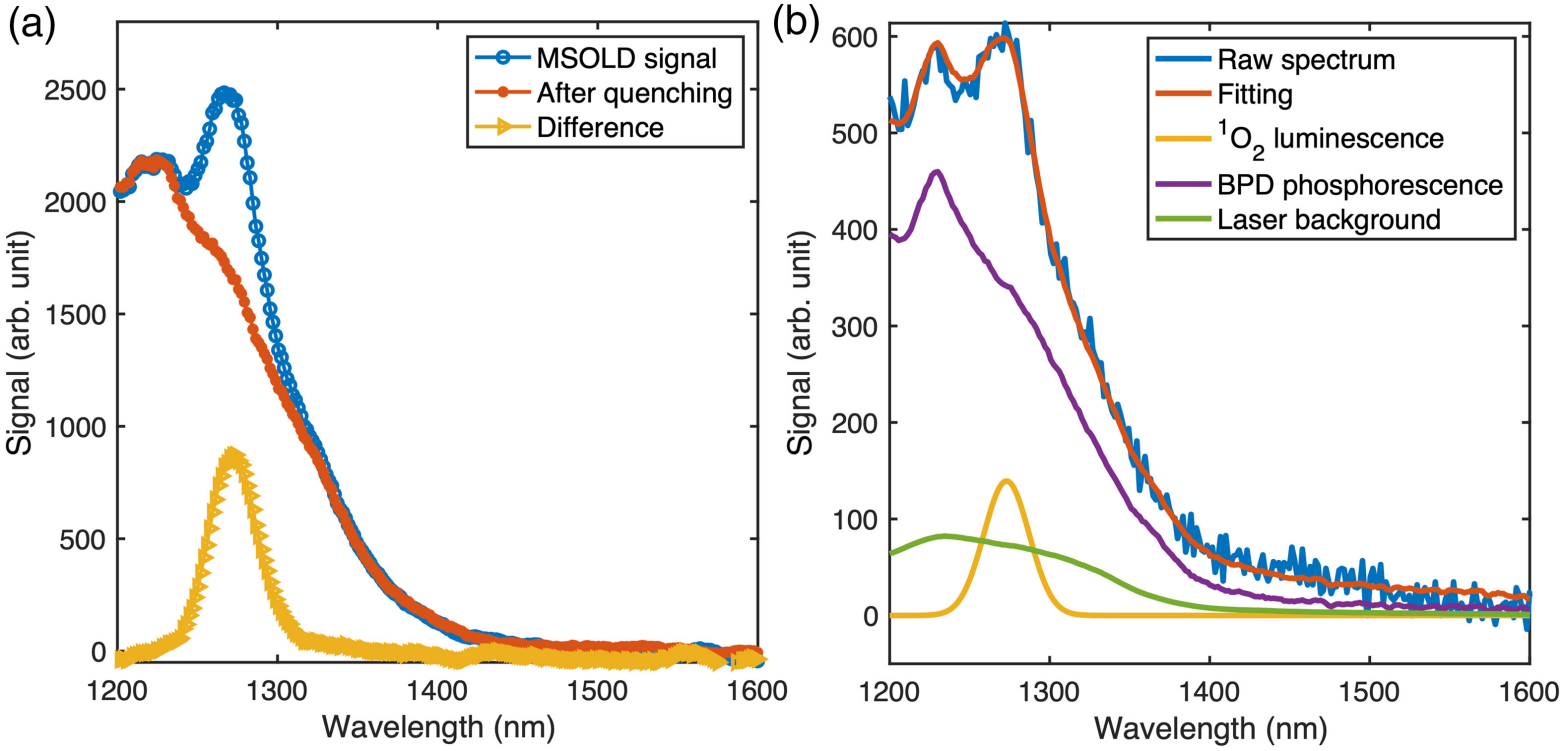
(a) Quenching of the MSOLD signal using sodium azide in a BPD solution prepared in pure methanol allows for the differentiation of singlet oxygen luminescence as the difference between the signals before and after quenching corresponds to the O12 luminescence. (b) SVD fitting of the measured spectrum to the basis functions.

Liquid phantoms of BPD concentrations 2, 3, 4, 5, or 6  mg/L were prepared. The NIR spectra were collected continuously using the Avantes spectrometer (AvaSpec–NIR256–1.7–EVO, Avantes, Lafayette, Colorado, United States) operating in low-noise mode with 900 s integration every 100 s during the entire ($765\;J/{\mathrm{cm}}^{2}$) PDT irradiation. Each spectrum was measured for 5 s. O12 luminescence, the BPD phosphorescence, and the laser background were extracted using the singular value decomposition (SVD) fitting algorithm, as illustrated in Fig. 2(b).

### 2.2 Singlet Oxygen Explicit Dosimetry

The BPD concentration in the liquid phantom during the PDT irradiation was monitored using a custom-built contact probe that measured the fluorescence around the 690 nm peak, excited by blue light (405±5  nm). Changes in this signal were used to track the BPD photobleaching throughout the experiments, taking measurements every 100 s with the treatment laser temporarily paused. The oxygen concentration in the phantom was similarly monitored using a phosphorescence-based oxygen probe (OxyLite Pro, Oxford Optronix, Oxford, United Kingdom), coupled with an oxygen bare-fiber sensor (NX-BF/OT/E, Oxford Optronix, Oxford, United Kingdom) positioned within the liquid phantom. The oxygen concentration was derived by applying a conversion factor of 1.295  μM/mmHg,^21^ as commonly done in tissue studies. Notably, the ground state oxygen concentration within the liquid phantom was initially measured at 194  μM and remained constant throughout the entire PDT, likely attributed to the fact that we are just measuring the signal for the surface layer within ∼2 to 3 mm^22^, and the oxygen diffusion from the surrounding air is very fast ($1.96\times 10^{-5}\;{\mathrm{cm}}^{2}\;s^{-1}$).^18^

The SOED model was used to calculate the concentration of singlet oxygen (O12) throughout the experiment. This macroscopic model employs a system of differential equations to describe the Type II PDT process, capturing the dynamic interactions among singlet oxygen concentration (${[}^{1}O_{2}]$), ground-state oxygen concentration ([O32]), fluence rate (ϕ), and photosensitizer concentration ($\left[S_{0}\right]$). The [O12] is calculated through the following equations.

[O12]=ξτΔ[O32][O32]+βϕ[S0]1(σ([S0]+δ)+1), (1)

d[S0]dt=−ξ[O32][O32]+βϕ[S0](σ([S0]+δ)(σ([S0]+δ)+1)), (2)

d[O32]dt=−ξ[O32][O32]+βϕ[S0](σ([S0]+δ)+k7[A]τΔ(σ([S0]+δ)+1)), (3)

[O12]rx=∫ξ[O32][O32]+βϕ[S0]1(σ([S0]+δ)+1)dt. (4)

The relevant parameters *in vitro* are outlined in Table 1. The oxidation of biomolecular acceptors, [A], is zero when there is no ${\mathrm{NaN}}_{3}$ in the MeOH solution. $k_{7}$ is the rate constant for O12 quenching by the substrate that can be determined experimentally by measuring the singlet oxygen lifetime. These parameters were sourced from literature,^16^ as listed in Table 1.

**Table 1:** Summary of photophysical parameters for BPD *in vitro*.^16^

Parameter	Definition	*In vitro*
β (μM)	Oxygen quenching threshold concentration	11.9
δ (μM)	Low concentration correction	33
ξ (${\mathrm{cm}}^{2}\;{\mathrm{mW}}^{-1}\;s^{-1}$)	Specific oxygen consumption rate	$(51\pm 15)\times 10^{-3}$
($\mu M^{-1}$)	Specific photobleaching ratio	$1.7\times 10^{-5}$
$\tau_{\Delta }$ (s)	Singlet oxygen lifetime	$(9.4\pm 0.2)\times 10^{-6}$
[A] (μM)	Oxidation of biomolecular acceptors	0

The measured near-infrared spectra were analyzed using an SVD algorithm implemented in MATLAB, based on three basis functions: (1) the singlet oxygen (O12) luminescence spectrum, representing the 1270 nm emission from the transition of excited triplet-state oxygen to the ground state; (2) the BPD phosphorescence, originating from the decay of triplet-state BPD to its ground state; and (3) the residual laser background from the laser source. The BPD phosphorescence background was measured using a BPD solution in clear methanol after quenching the O12 luminescence with sodium azide (${\mathrm{NaN}}_{3}$). The laser background was determined by directing the laser light directly into the optical fiber coupled to the Avantes spectrometer (AvaSpec–NIR256–1.7–EVO, Avantes, Lafayette, Colorado, United States), in the absence of any photosensitizer. The effect of BPD photobleaching was confirmed using a custom-made multifiber contact probe and a multichannel CCD spectrograph (WinSpec, Teledyne Princeton Instruments, Trenton, New Jersey, United States) under 405±5  nm laser excitation (IQ1C05, Power Technology Inc., Little Rock, Arkansas, United States) to capture the BPD fluorescence spectra.

## 3 Results

Figure 2(a) illustrates the quenching of the singlet oxygen (O12) luminescence using sodium azide (${\mathrm{NaN}}_{3}$; Thermo Fisher Scientific, Waltham, Massachusetts, United States) in a solution of BPD dissolved in clear methanol. The blue curve shows a distinct O12 emission peak at 1270 nm before quenching. Upon addition of ${\mathrm{NaN}}_{3}$, the O12 luminescence is significantly reduced, as shown in the red curve. A small residual signal remains, reflecting the challenge of eliminating O12 emission. The yellow curve represents the difference between the original MSOLD spectrum and the postquenching spectrum, corresponding to the extracted O12 luminescence. Figure 2(b) presents an example of spectral decomposition using the SVD method. The raw MSOLD spectrum is decomposed into three primary components: the O12 luminescence, BPD phosphorescence, and the laser background. These components are shown individually in the figure. This decomposition approach was applied consistently across all spectral analyses in this study.

**Fig. 3:**
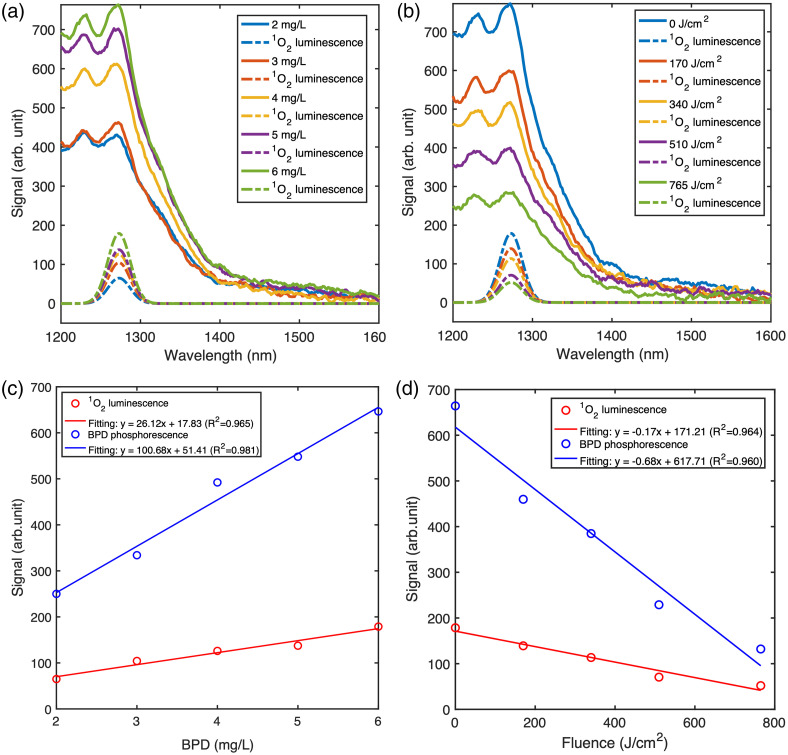
(a) Singlet oxygen (O12) spectra (solid lines) and extracted O12 components (dashed lines) from SVD fitting in a liquid phantom with BPD concentrations ranging from 2 to 6 before PDT irradiation. (b) O12 spectra (solid lines) and corresponding O12 components (dashed lines) at 0, 170, 340, 510, and $765\;J/{\mathrm{cm}}^{2}$ during PDT irradiation in a liquid phantom with a BPD concentration of 6  mg/L. (c) The O12 luminescence and corresponding BPD phosphorescence as a function of BPD concentration from panel (a), with linear fits displayed. (d) The O12 luminescence and corresponding BPD phosphorescence as a function of PDT irradiation time from panel (b), with linear fits displayed.

The solid lines in Fig. 3(a) represent the five-point-smoothed measured MSOLD spectra, whereas the dashed lines indicate the corresponding extracted O12 luminescence. It is expected that higher BPD concentrations yield stronger MSOLD spectra. As shown in Fig. 3(c), both the O12 luminescence and BPD phosphorescence increase approximately linearly with drug concentration in the range of 2 to 6  mg/L. However, the rate of increase in O12 luminescence is notably smaller than that of BPD phosphorescence. This discrepancy may be attributed to effects such as self-quenching or oxygen depletion at higher BPD concentrations, which can limit the efficiency of singlet oxygen generation despite increased photosensitizer availability.

During the entire irradiation, the O12 luminescence falls due to photosensitizer photobleaching, as seen in Fig. 3(b), which shows the raw MSOLD spectrum and extracted O12 luminescence for a liquid phantom with 6  m/kg BPD at 0, 170, 340, 510, and $765\;J/{\mathrm{cm}}^{2}$ into the irradiation at an excitation fluence rate of $850\;\mathrm{mW}/{\mathrm{cm}}^{-2}$. Figure 3(d) shows the corresponding O12 luminescence and BPD phosphorescence as a function of PDT irradiation time, with linear fitting shown in the legend. Both the O12 luminescence and BPD phosphorescence decline approximately linearly with time, but BPD phosphorescence is decreasing faster than the O12 luminescence.

**Fig. 4:**
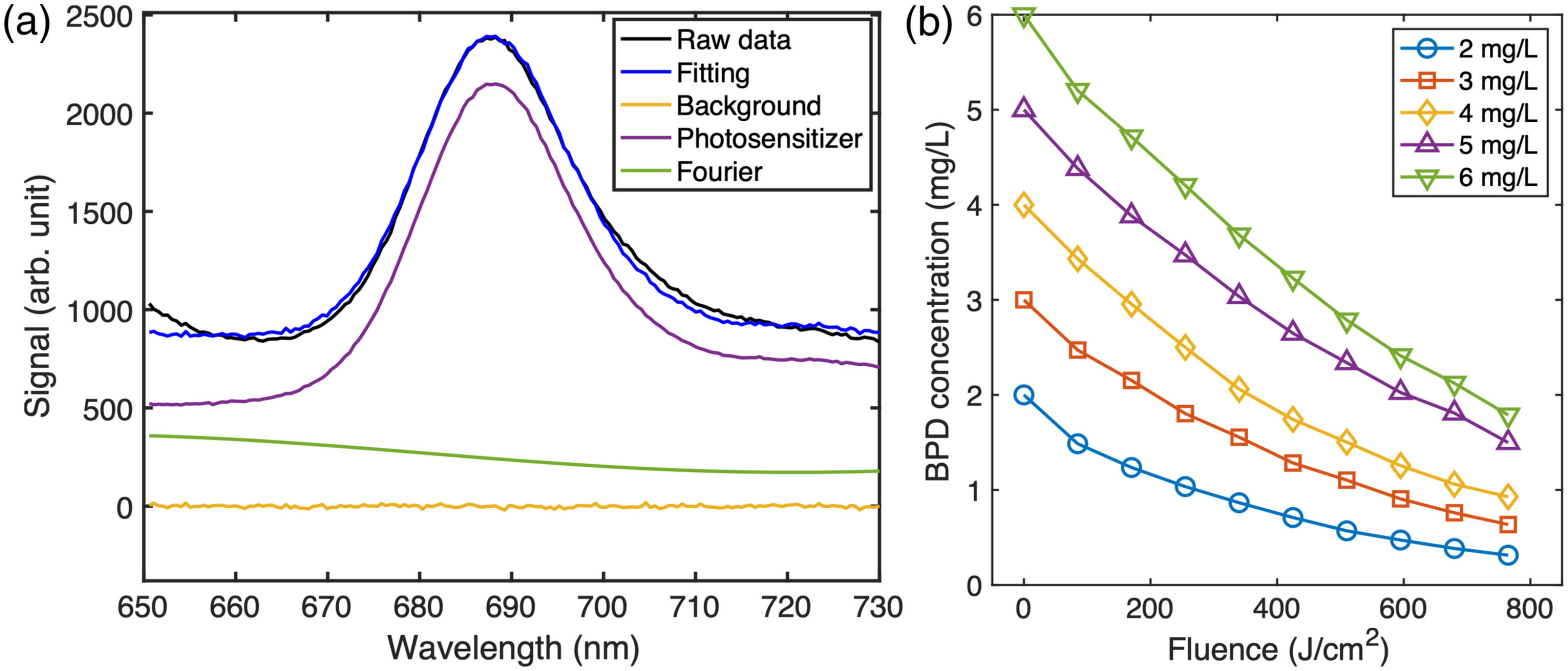
(a) BPD fluorescence at 690 nm excited by a blue light (406 nm) and the corresponding SVD fitting with photosensitizer fluorescence, background signal, and Fourier component. (b) Photobleaching of the BPD concentration and the corresponding fitting over 900 s of PDT irradiation for the five BPD concentrations.

Figure 4(a) shows the fluorescence spectra before the PDT with an initial BPD concentration of 6  mg/L BPD. The corresponding BPD concentrations at 0, 170, 340, 510, and $765\;J/{\mathrm{cm}}^{2}$ for phantoms with 2 to 6  mg/L BPD during PDT are plotted in Fig. 4(b). BPD phosphorescence decreases approximately linearly throughout the entire PDT, indicating a corresponding linear decrease in BPD concentration during the experiment.

**Fig. 5:**
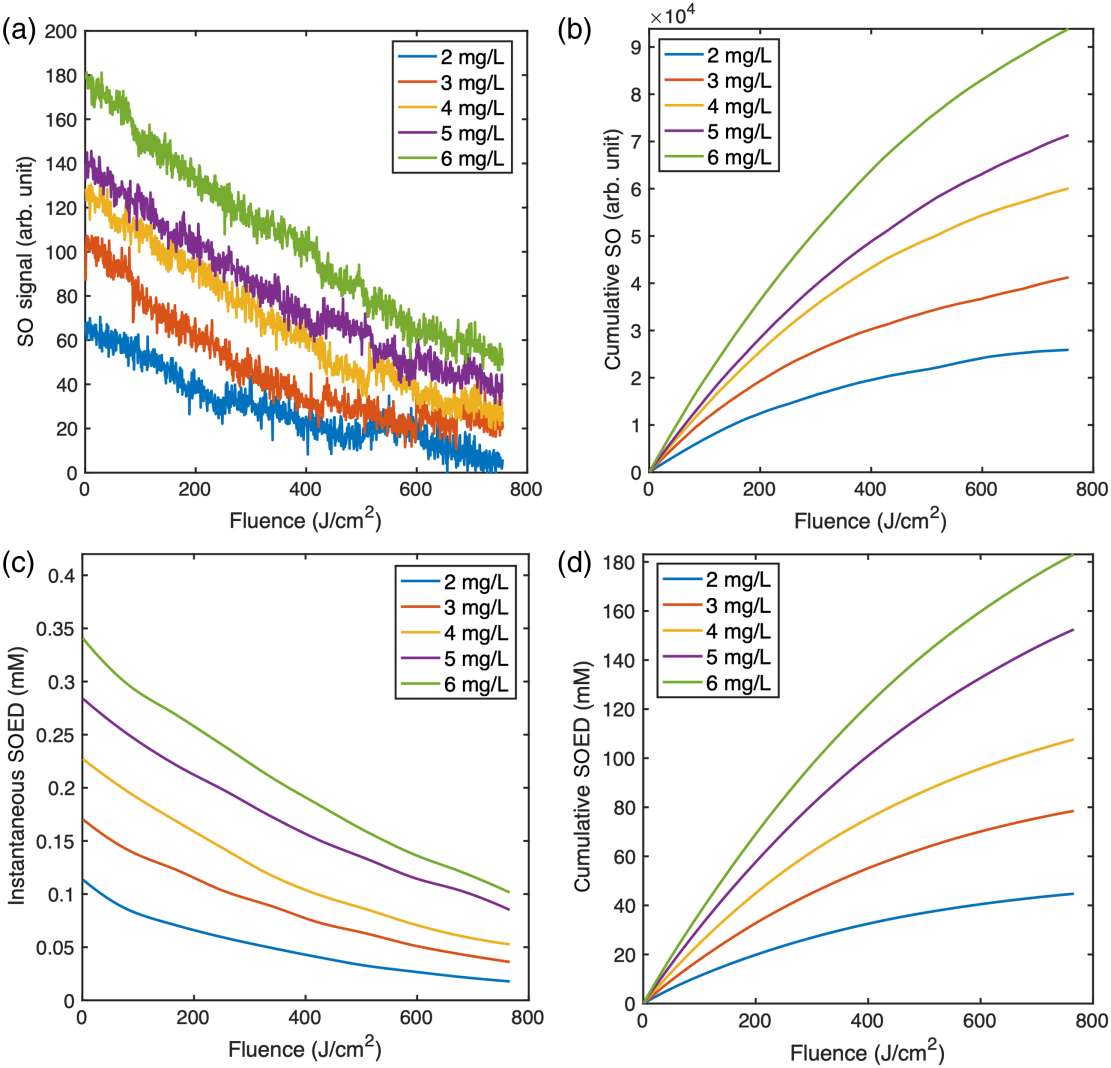
(a) Extracted instantaneous singlet oxygen (O12) luminescence over a $765\;J/{\mathrm{cm}}^{2}$ irradiation for a liquid phantom with BPD concentrations ranging from 2 to 6  mg/L. (b) Corresponding cumulative O12 luminescence over time for the data shown in panel (a). (c) Instantaneous singlet oxygen dosimetry (SOED) over the entire irradiation for a liquid phantom with BPD concentrations ranging from 2 to 6  mg/L. (d) Corresponding cumulative SOED concentration over time for the data shown in panel (c).

Figure 5(a) shows the extracted O12 luminescence during the entire PDT. The observed decrease in the O12 signal over fluence is primarily due to the decrease in BPD concentration and possibly oxygen depletion and a change of optical properties. The signals are noisier at lower concentrations due to the limited O12 luminescence, leading to a lower signal-to-noise ratio. Figure 5(b) presents the cumulative O12 luminescence, again increasing with initial BPD concentration. However, the measured cumulative O12 signal also depends on the experimental setup. Therefore, it does not provide a direct measure for PDT dosimetry that can be universally applied to determine the PDT dose.

Figure 5(c) illustrates the calculated instantaneous SOED over time for five different BPD concentrations. Figure 5(d) illustrates the cumulative SOED for the five BPD concentrations depicted in Fig. 5(c), where SOED is increasing with rising BPD concentration. The fitted slopes for instantaneous and cumulative SOED and MSOLD show slight differences, which may be attributed to methanol evaporation leading to a loss of the MSOLD signal. However, both the instantaneous and cumulative SOED exhibit a similar trend to the O12 luminescence, highlighting the consistency between the two measurement techniques.

**Fig. 6:**
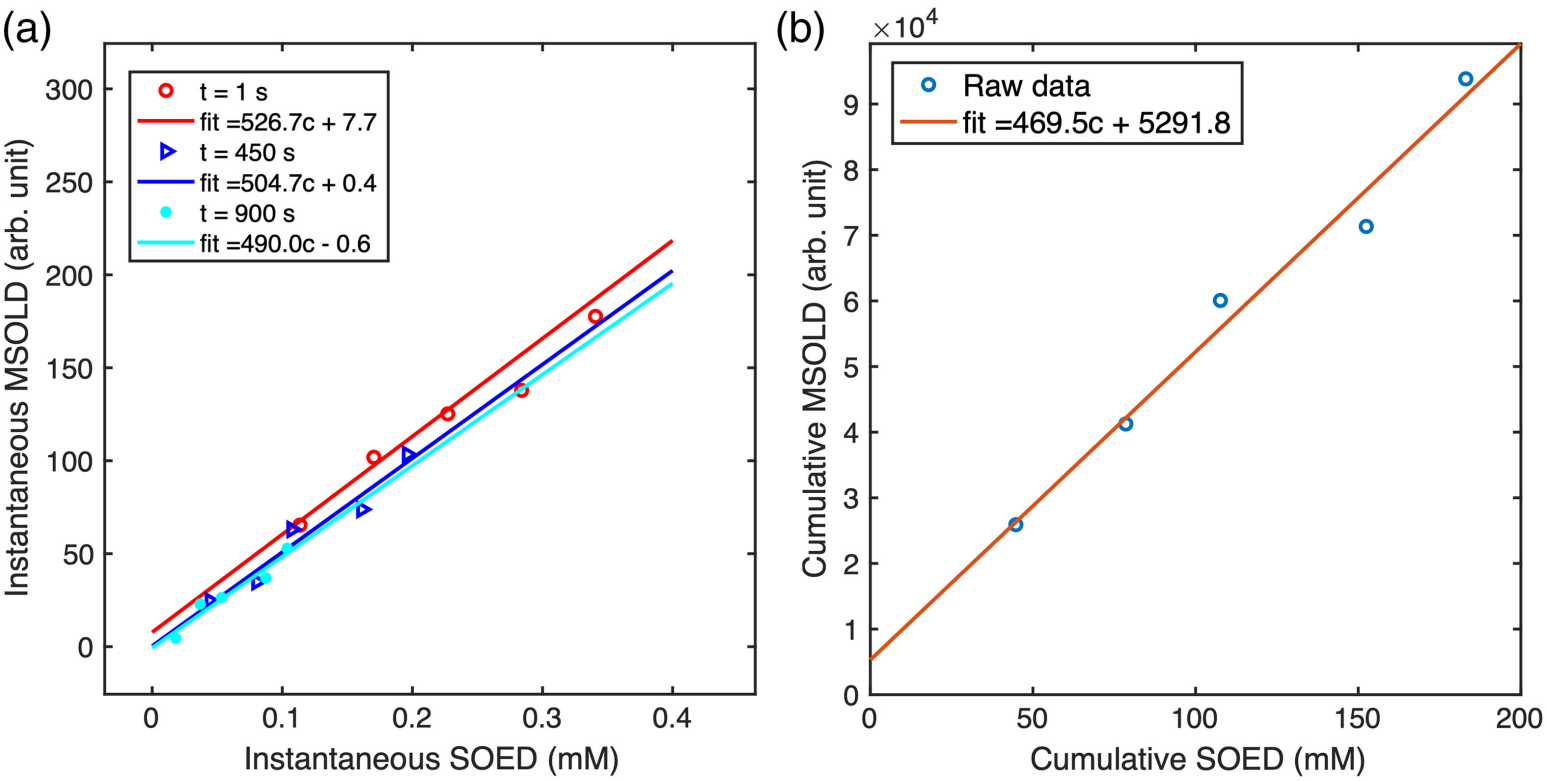
(a) Correlation between instantaneous singlet oxygen dosimetry (MSOLD) and instantaneous SOED at different time points (1, 450, and 900 s) during PDT irradiation, with linear fits shown for each time point. (b) Relationship between cumulative MSOLD and cumulative SOED, demonstrating a strong linear correlation, with the best-fit equation indicated in the legend.

Figure 6(a) compares the instantaneous SOED and instantaneous MSOLD at 0, 450, and 900 s across different BPD concentrations. Linear fits were obtained for all BPD concentrations at these time points, showing a consistent trend with only minor experimental variations. Correlations between MSOLD and SOED were evaluated using linear regression analysis. The deviations between instantaneous MSOLD and SOED are visible in the fittings at 1, 450, and 900 s, where the slopes are not identical, varying from 490 to 526.7, due to experimental variability. There is also a slight nonzero y-axis intercept in both the instantaneous and cumulative MSOLD–SOED comparisons, which is attributed to imperfections in the experimental data. However, we repeated these experiments multiple times with different drugs, and the results consistently demonstrated the linear correlation between MSOLD and SOED, underscoring the robustness of our conclusion. Notably, the fittings at different time points exhibit nearly identical slopes, indicating strong reproducibility. Figure 6(b) presents the comparison between cumulative SOED and cumulative MSOLD, with a linear fit demonstrating a strong correlation between the two metrics. The similar slopes of the fitting lines for both instantaneous and cumulative MSOLD and SOED further highlight the reliability of MSOLD for PDT applications. The strong agreement between MSOLD and SOED measurements confirms the accuracy of both techniques.

Because absorption and scattering can significantly affect both MSOLD and SOED signals, the CFs are necessary to account for variations in the detected singlet oxygen (O12) luminescence. We prepared 25 distinct liquid phantoms with absorption coefficients ($\mu_{a}$) ranging from 0.1 to $1.0\;{\mathrm{cm}}^{-1}$ and reduced scattering coefficients ($\mu_{s}^{\prime }$) ranging from 5 to $40\;{\mathrm{cm}}^{-1}$ at 690 nm. Black India ink was used to simulate tissue absorption, whereas Nutrilipid was used to simulate scattering properties. The BPD concentration was fixed at 25  mg/L, which had an absorption coefficient of $2.24\;{\mathrm{cm}}^{-1}$ at 690 nm. We used the absorption coefficients of black India ink to model the liquid phantom, isolating the optical effects from BPD absorption.

**Fig. 7:**
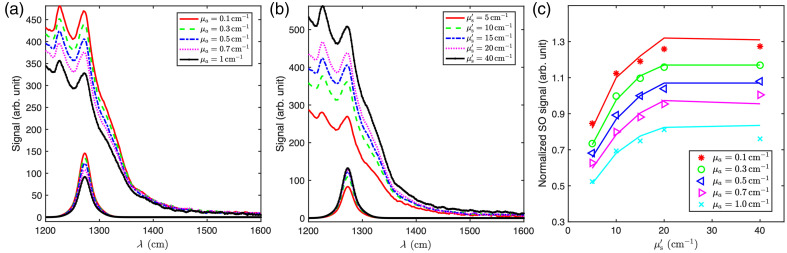
(a) MSOLD signals and the corresponding extracted O12 luminescences obtained through SVD fitting for liquid phantoms with varying absorption coefficients ($\mu_{a}=0.1$ to $1.0\;{\mathrm{cm}}^{-1}$), excluding BPD absorption. (b) MSOLD and extracted O12 signals for phantoms with varying reduced scattering coefficients ($\mu_{s}^{\prime }=5$ to $40\;{\mathrm{cm}}^{-1}$). (c) O12 signal (points) and corresponding Monte Carlo simulations (solid lines) for different optical properties, normalized to the results with a $\mu_{a}=0.3\;{\mathrm{cm}}^{-1}$ and $\mu_{s}^{\prime }=10\;{\mathrm{cm}}^{-1}$ that approximates the typical optical properties of pleural tissue.^23^

Figure 7(a) illustrates the influence of varying $\mu_{a}$. As expected for the diffuse-reflectance geometry used, both MSOLD and SOED signals decrease as $\mu_{a}$ increases, whereas the scattering increases the local fluence rate in superficial layers of the phantom and produces higher O12 luminescence. In Fig. 7(c), the experimental results are compared with Monte Carlo simulations as described in a previous paper.^20^

To account for the influence of varying tissue optical properties, we derived correction factors for each combination of $\mu_{a}$ and $\mu_{s}^{\prime }$, as summarized in Table 2. To obtain these factors, we measured and simulated the detected singlet oxygen signal with various tissue optical properties and then compared it to a reference point ($\mu_{s}^{\prime }=10\;{\mathrm{cm}}^{-1}$, $\mu_{a}=0.3\;{\mathrm{cm}}^{-1}$). The ratio between the measured/simulated signal and the reference provides the correction factor, ensuring that the measured singlet oxygen signal is not biased by variations in tissue optical properties. Notably, the correction factors obtained for BPD-mediated PDT closely resemble those reported previously for Photofrin-mediated PDT.^20^

**Table 2:** Correction factors for singlet oxygen luminescence under various $\mu_{a}$ and $\mu_{s}^{\prime }$ of phantoms.

$\mu_{s}^{\prime }$ (${\mathrm{cm}}^{-1}$)	$\mu_{a}$ (${\mathrm{cm}}^{-1}$)				
	0.1	0.3	0.5	0.7	1.0
5	1.19	1.33	1.48	1.62	1.89
10	0.88	1.00	1.12	1.23	1.43
15	0.80	0.87	0.97	1.08	1.25
20	0.74	0.84	0.91	1.00	1.18
40	0.74	0.83	0.90	1.02	1.17

## 4 Discussion

Here, we systematically compared the O12 luminescence measured by MSOLD with the concentration predicted by SOED using Visudyne^®^ (BPD) in methanol. We recognize that this does not fully replicate the physiological conditions of real tissue, particularly in terms of oxygen concentration and distribution and optical heterogeneity. Specifically, the oxygen concentration in the phantom is significantly higher than what is typically found in tumors. As a result, oxygen is not a limiting factor for O12 generation in the phantom model, whereas in real tumors, hypoxia can substantially reduce O12 production.

Despite these differences, our results demonstrate a strong correlation between MSOLD-measured O12 luminescence for low BPD concentration from 2 to 6  mg/L and the O12 concentration estimated by SOED. However, the O12 luminescence alone is insufficient to determine the absolute singlet oxygen concentration due to the effects of spectrometer performance, optical fiber geometry, and the tissue optical properties of phantoms. Therefore, we investigated the impact of tissue optical properties on singlet oxygen luminescence detection and derived correction factors to compensate for signal deviations caused by variations in optical properties.

MSOLD offers the advantage of direct, real-time monitoring of O12 production during PDT. It is also a faster and simpler method than SOED, requiring measurement of only one parameter, with no need for complex modeling or postprocessing. However, MSOLD faces significant challenges, particularly *in vivo*, due to the extremely weak O12 luminescence signal (∼1270  nm). This signal can be easily overwhelmed by stronger background emissions, such as BPD phosphorescence and laser scattering. Therefore, improving the signal-to-noise ratio is critical to ensure reliable detection of the O12 luminescence. The detected MSOLD signal also depends on several experimental factors, including the detection fiber diameter, spectrometer sensitivity, the angle between the detection fiber and the laser, the distance from the fiber tip to the phantom, and the coupling efficiency between the fiber and the spectrometer. Therefore, calibrating the detected O12 luminescence signal is essential to determine the absolute O12 concentration.

By contrast, the SOED model provides a more comprehensive and quantitative estimation of absolute O12 concentrations by incorporating drug concentration, oxygen concentration, and laser fluence rate—parameters that can generally be measured with higher reliability than the weak O12 luminescence signal. However, SOED has its own limitations: measuring the drug concentration often requires interrupting the PDT as the wavelength of both BPD phosphorescence and laser is 690 nm as measuring drug concentration relies on detecting BPD phosphorescence at 690 nm excited by a blue light source. Because BPD-mediated PDT also uses a 690 nm laser, there would be interference between the treatment laser and the luminescence signal. Therefore, the PDT process must be briefly paused to measure the BPD concentration accurately. The need to quantify three separate parameters introduces additional sources of error. Moreover, SOED calculations are typically performed after PDT irradiation, preventing real-time feedback.

Combining MSOLD and SOED leverages the strengths of both: real-time monitoring from MSOLD and quantitative dose assessment from SOED. Another emerging approach is the use of an isotropic detector for measuring O12 luminescence, which has the potential to determine absolute O12 concentrations with appropriate calibration. The signal detected using a flat-cut fiber is angle-dependent, making it difficult to obtain consistent results in clinical applications. By contrast, the signal measured by an isotropic detector is independent of the fiber’s orientation. Isotropic detectors are already used clinically for measuring laser fluence rates with appropriate calibration. Therefore, we believe that, with proper calibration, the isotropic detector has strong potential for determining absolute singlet oxygen concentrations. However, this method remains technically challenging as the detected O12 signal from isotropic probes is typically only about one-third of that collected by standard optical fibers.^24^

Together, combining both MSOLD and SOED techniques offers a synergistic strategy for accurate, real-time dosimetry and improved monitoring of PDT efficacy.

## 5 Conclusion

In this study, we investigated the direct detection and quantification of O12 near-infrared luminescence during photodynamic therapy (PDT) using MSOLD under 900 s of laser irradiation in a liquid phantom model with varying concentrations of BPD. The corresponding SOED values were calculated based on measured laser fluence rate, BPD concentration, and ground-state oxygen levels. We demonstrated a linear correlation between MSOLD and SOED for BPD concentrations ranging from 2 to 6  mg/L, confirming the consistency between these two approaches for quantifying O12 during PDT. The results demonstrate a promising approach for directly measuring singlet oxygen concentration in real time during PDT, which could significantly improve the accuracy and effectiveness of photodynamic therapy.

In addition, we showed that tissue optical properties significantly impact the accuracy of O12 luminescence detection. To address this, we developed correction factors to adjust MSOLD measurements for clinically relevant optical properties, thereby enhancing accuracy. Given its ability to provide real-time feedback, as well as its simplicity and cost-effectiveness, MSOLD offers a promising tool for clinical PDT dosimetry.

## Data Availability

Data underlying the results presented in this paper may be obtained from the authors upon reasonable request.
